# Protein-Based Graphene Biosensors: Optimizing Artificial Chemoreception in Bilayer Lipid Membranes

**DOI:** 10.3390/membranes6030043

**Published:** 2016-09-07

**Authors:** Christina G. Siontorou, Konstantinos N. Georgopoulos, Georgia-Paraskevi Nikoleli, Dimitrios P. Nikolelis, Stefanos K. Karapetis, Spyridoula Bratakou

**Affiliations:** 1 Laboratory of Simulation of Industrial Processes, Department of Industrial Management and Technology, School of Maritime and Industry, University of Piraeus, Piraeus 18534, Greece; csiontor@unipi.gr (C.G.S.); kosnosgeo@gmail.com (K.N.G.); 2 Laboratory of Inorganic & Analytical Chemistry, School of Chemical Engineering, Department of Chemical Sciences, National Technical University of Athens, Athens 15780, Greece; tzwrtzia85@hotmail.com (G.-P.N.); stevekara@chem.uoa.gr (S.K.K.); mar05059@marine.aegean.gr (S.B.); 3 Laboratory of Environmental Chemistry, Department of Chemistry, University of Athens, Athens 15771, Greece

**Keywords:** biosensors, electrochemistry, graphene nanosheets, ZnO nanowalls, stabilized lipid films, cholesterol oxidase

## Abstract

Proteinaceous moieties are critical elements in most detection systems, including biosensing platforms. Their potential is undoubtedly vast, yet many issues regarding their full exploitation remain unsolved. On the other hand, the biosensor formats with the higher marketability probabilities are enzyme in nature and electrochemical in concept. To no surprise, alternative materials for hosting catalysis within an electrode casing have received much attention lately to demonstrate a catalysis-coated device. Graphene and ZnO are presented as ideal materials to modify electrodes and biosensor platforms, especially in protein-based detection. Our group developed electrochemical sensors based on these nanomaterials for the sensitive detection of cholesterol using cholesterol oxidase incorporated in stabilized lipid films. A comparison between the two platforms is provided and discussed. In a broader sense, the not-so-remote prospect of quickly assembling a protein-based flexible biosensing detector to fulfill site-specific requirements is appealing to both university researchers and industry developers.

## 1. Introduction

**Figure 1:**
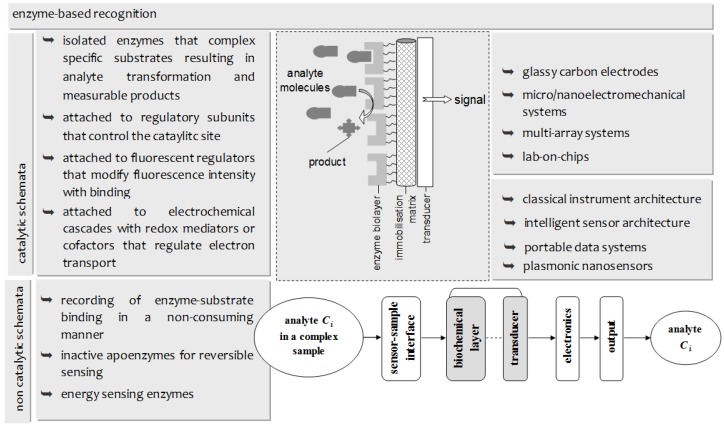
Overview of the basic concepts and the technology evolution of enzyme-based biosensing platforms.

The interplay between proteinaceous moieties and analytes in natural chemoreception has undoubtedly paved the biosensor trajectory over the last 30 years [1]. The seizing, however, of nature’s selectivity, specificity, and sensitivity, and its development into a device, proved a difficult task in a variety of ways. Protein functioning ex vivo is not merely an environmental issue; nature relies on a multi-faceted and multilevel regulatory network, including gene expression, hormonal production, and a variety of biochemical interactions in order to assure adequate function [2,3]. In order to compensate, research focused towards two aspects (Figure 1): materials, leading to fluorescent tags [4], catalysis regulators [5], redox mediators [6], and apoenzymes [7], as well as transducers, ranging from classical device architectures to intelligent sensor systems [8]. Despite this, certain drawbacks remain, mainly in coupling effectively proteins with transducers and in relaying reliably the biochemical information to an output screen [9,10,11]. These problems have to be solved at two scales: the micro-scale (biolayer/transducer interface) and the macro-scale (sensor system) [1,2].

The simplest and most effective means to confine proteins on the surface of a transducer involve either lipid bilayer films that adsorb the protein moieties [12,13] or permselective membranes that enclose the biolayer [14]. While the former offers a clear thermodynamic advantage for optimising the biochemical reaction [1,13], the latter provides (some degree of) protection to interference and leakage [9,10]. The issue of orientation becomes more critical in the affinity-based systems [4,9]. Exact deposition procedures emerged using a variety of ligands and ligation methods that lead to rigid structures with simple or more complicated architectures [10]; sensor construction became an exact multi-step procedure or a costly equipment-intensive protocol, whereas sensor regeneration recipes could be demanding and ineffective [15].

Human immunoglobulin adsorption has been recently investigated by means of quartz crystal microbalance dissipation, atomic force microscopy, surface acoustic wave, and surface plasmon resonance techniques [16]. Angelova et al. [17] used X-ray diffraction to study lipid cubic phases as stable nanochannel network structures for protein biochip development.

At the macro-domain, sensor design has proved equally demanding. Catalytic and affinity events cannot share the same transduction technology [3,8]. In enzyme-based recognition, a rapid reaction takes place between the enzyme and the analyte leading to the elution of products and the consumption of the substrate. Signal amplification is almost inherent and a large amount of chemical information passes to the sensing layer giving large signals. Not surprisingly, a large body of lab-developed biosensors are catalytic, taking advantage of the enzyme-substrate specificity and the high sensitivity of measurements [1]. In complex samples, however, the interaction of sample components with the transducer or with the protein immobilization layer cannot be ruled out [2]. On the other hand, antibody-based biosensors have evolved to high specificity systems but still need some sort of follow-up signal amplification, either engineered (florescent tags or enzyme bioconjugates) or electronic (signal processing and imaging software) [4].

Notwithstanding, the marketability of the devices remains low, mainly due to the lack of sensor stability studies and the inherent (limited by enzyme activity) short lifetimes under operation [1]. Attempts to overcome the marketability hurdles have been concentrated on the testing of new materials that could prove more suitable to handle the underlying biosensing science and deliver a reliable device. Graphene-based nanomaterials have added a new tool in biosensor research, offering amply unique capabilities in fast electron wiring, high thermal conductivity, excellent mechanical stability, adequate biocompatibility, and a tremendously large surface-area-to-volume ratio that could boost device commercialisation [14,18,19,20]. The high electrocatalytic activity of graphene towards peroxide was found to enhance the performance of oxidase-based biosensors [18]; the activity of the enzyme was fully retained and the sensitivity was adequately increased to allow for considerably lower detection limits than conventional platforms. Graphene sheets carry defects at their edges that could serve in electron wiring between the sheet and its substrate [19,20]; thus, well-defined and resolved oxidation peaks of ascorbic acid, dopamine, and uric acid have been recorded. Graphene oxide provides a beneficial adsorption matrix for antibodies and proteins via oxygen-mediated amide condensation reactions or electrostatic interactions [14,21]. Graphene oxide has been recently utilised as a nanocarrier for the signal amplification enzyme cascade in an electrochemical immunosensor for phosphorylated protein [22]; the detection limit achieved was 10 times lower than that of the conventional immunosensor.

On the other hand, ZnO nanowires, nanorods, nanoflowers, and nanotubes exhibit, also, a high surface-to-volume ratio, along with a non-toxic potential, optical transparency, high ionicity, good biocompatibility, and high electron communication features [23]. Interestingly, ZnO with a high isoelectric point (IEP ~ 9.5) might be suitable for the adsorption of low IEP proteins or enzymes; a positively-charged ZnO nanorods matrix provided a friendly microenvironment for negatively-charged proteins and enzymes, whereas they promoted the direct electron transfer between the enzymes and the electrode to a large extent [24]. Zhang et al. [25] immobilized uricase on ZnO nanorods for a reagentless uric acid biosensor. Further, one-dimensional ZnO nanostructures can be easily converted to biosensor platforms due to their fast vertical growth on a variety of substrates [26], and their high stability at the neutral pH range [27,28,29,30]. A sonochemical approach for rapid growth of ZnO nanowalls was recently published and could be used for helium and ammonia sensing [31]. ZnO-based biosensors have been proposed for the detection of proteins, bivalent ions, glucose, and phenolic compounds [32,33,34,35,36].

Both nanostructures are presented as ideal materials to modify electrodes and biosensor platforms, especially in protein-based detection. Our group developed graphene-based and ZnO nanowall biosensor platforms for the electrochemical detection of cholesterol, based on cholesterol oxidase incorporated in stabilised lipid films. A comparison between the two platforms is provided here below.

## 2. Materials and Methods

### 2.1. Reagents and Solutions

Cholesterol oxidase (EC 1.1.3.6, 28 U·mg^−1^) (Sigma Aldrich, St. Louis, MO, USA), cholesterol (Sigma Aldrich), dipalmitoyl phosphatidylcholine (C16:0) (DPPC) (Sigma Aldrich), Methacrylic acid (Aldrich-Chemie), ethylene glycol dimethacrylate (Aldrich-Chemie), and 2,2′-azobis-(2-methylpropionitrile) (AIBN) (Merck) were used for the preparation of the lipid mix solution without any further purification. Double-distilled water was used for all experiments and a Milli-Q cartridge filtering system (Milli-Q, Millipore, El Paso, TX, USA) was utilized to obtain the water with a minimum resistivity of 18 MΩ. Equimolar concentrations (0.05 M) of KH_2_PO_4_ and Na_2_HPO_4_, in NaCl (0.9%) electrolyte, were mixed together to prepare the phosphate-buffered solutions (PBS). Cholesterol stock solution was prepared in water (50 mL) containing 2% (*v/v*) 2-propanol and 2% (*v/v*) Triton X-100 in a bath at 60 °C to avoid precipitation. Cholesterol oxidase (initial concentration of 500 U·mL^−1^) was diluted with 10 mM of tris-HCl buffer solution. All enzyme solutions were refrigerated (4–8 °C) when not in use. Glass microfiber filters (Whatmam Scientific Ltd., Kent, UK) with nominal pore sizes 0.7 and 1.0 mm were used as lipid film supports. All other reagents were of analytical range and supplied by Sigma Aldrich.

### 2.2. Procedures

Stabilized lipid films were prepared by polymerization, as previously described in literature [37,38,39]; the lipid (5 mg of DPPC) was mixed with methylacrylic acid (0.070 mL), ethylene glycol dimethacrylate (0.8 mL), 2,2′-azobis-(2-methylpropionitrile (8 mg), and acetonitrile (1.0 mL). The mixture was purged with nitrogen for 1 min and then sonicated for 30 min; 0.15 mL of this mixture was spread on the microfilter. The filter with the mixture was then irradiated using a UV deuterium lamp. The duration of polymerization was four hours. Alternatively, thermal polymerization in 80 °C could be used, but the duration of the reaction is prolonged. These membranes could be stored for more than two months at room temperature and conditions (25 °C, 65% relative humidity) [39].

Cholesterol oxidase (15 mL of enzyme suspension) was spread on the microfilter after the addition of the lipid solution; thus, the enzyme was incorporated into the lipid film during its formation.

ZnO nanowall electrodes have been synthesised in situ on aluminum (Al) foil (size 1 cm × 1 cm) using equimolar concentrations (50 mM) of zinc nitrate hexahydrate (Zn(NO_3_)_2_·6H_2_O) and hexamethylenamine (C_6_H_12_N_4_) using the sonochemical protocol of Nayak et al. [31] and the Scotch tape method described in [40].

The graphene electrode has been prepared as previously described [12,41]. The working bioelectrode has been assembled as follows: graphene dispersion (ca. 0.4 mg/mL) in *N*-methyl-pyrrolidone (NMP) has been homogenised through mild sonication for 180 h, followed by centrifugation at 700 rpm for 2 h [31]. The resultant has been transferred onto a copper wire (with a diameter of 0.25 mm) mounted on a glass fibre filter carrying the enzyme-contained lipid film. The organic solvent has been evaporated using a fan heater.

**Figure 2:**
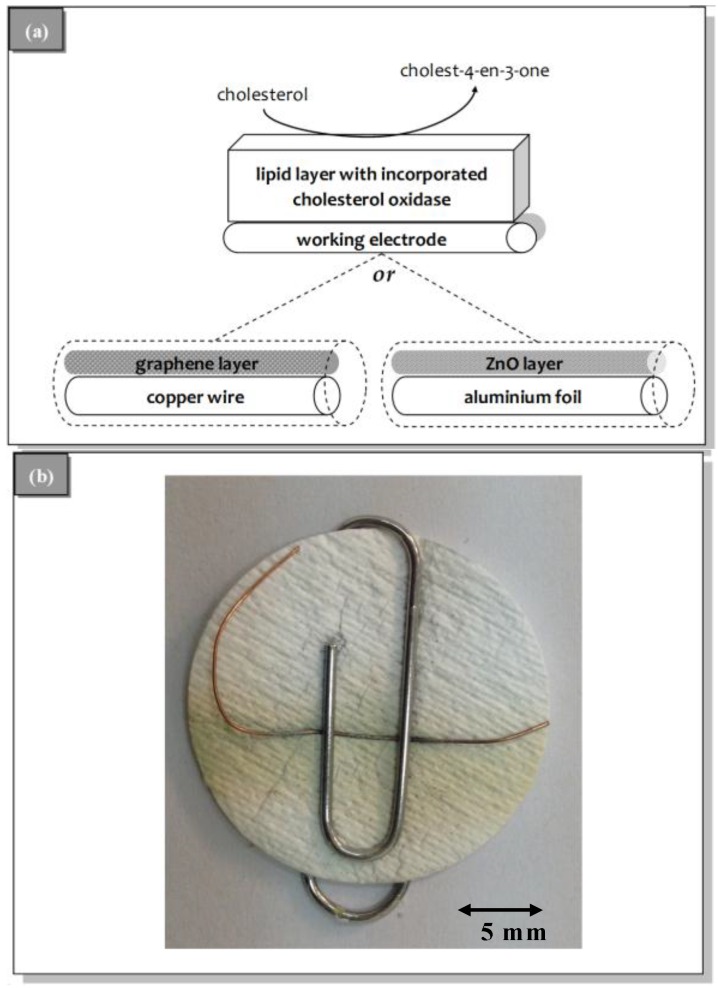
(**a**) Schematic of the experimental set-up and the two alternative bioelectrode surfaces: graphene nanosheets (left) or ZnO nanowalls (right); and (**b**) a photo of the microfiber filter with the deposited lipid film.

The preparation of the potentiometric biosensor concluded after the encapsulation of the filter-supported polymerized lipid film with incorporated cholesterol oxidase onto the wire containing the ZnO nanowalls or graphene nanosheets electrodes (Figure 2).

### 2.3. Electrochemical Measurements

A two-electrode system, i.e., the working bioelectrode: a stabilized polymeric lipid membrane coupled to graphene or ZnO nanowalls, and the reference electrode: a standard Ag/AgCl, used to measure the potentiometric electrochemical response of the sensor using a Keithley Electrometer Model 614 (in the voltage mode, Beaverton, OR, USA); the voltage was measure against the reference electrode [39]. Potentiometric detection was based on the redox potential caused by oxygen/hydrogen peroxide ratio shifts.

Sensor calibration performed in a stopped-flow mode: cholesterol solutions, at 10-μL or 20-μL aliquots, were injected into the carrier electrolyte (pH = 7.4) inflow; the flow stopped for 5 min (adequate time to record the response) and then re-started for sensor regeneration.

### 2.4. Sensor Validation for Biological Samples

Generally, a biosensor is considered suitable for practical applications if it exhibits adequate selectivity of target analytes over other sample constituents. Aiming at blood sample analyses, interference studies performed using a competitive study, i.e., testing cholesterol and interferent together in solution: the compounds examined were: maltodextrin (100 + 1), dextrose (100 + 1), fructofuranose (100 + 1), ascorbic acid (100 + 1), lactose (10 + 1), sorbitol (10 + 1), mannitol (10 + 1), glucose (10 + 1), leucine (10 + 1), carboxymethylcellulose (1 + 1), glycine (10 + 1), calcium phosphate (1 + 1), sodium tartrate, citric acid, sodium bicarbonate (10 + 1), sodium benzoate (10 + 1), calcium acetate (10 + 1), caffeine (10 + 1), urea (100 + 1), uric acid (1 + 1), creatinine (1 + 1), and aspartame (10 + 1). Protein interference has been also investigated using bovine serum albumin (buffered) solution at the concentration range usually found in human serum (6%–8% *m/v*).

## 3. Results and Discussion

Regardless of electrode type, the cholesterol oxidase sensors developed herein exhibited higher sensitivity towards cholesterol (57 and 64 mV per decade of cholesterol concentration for the ZnO-based and the graphene-based systems, respectively) as compared to other biosensor platforms reported in literature. For example, the electrochemical cholesterol oxidase biosensor based on ZnO nanorods proposed by Israr et al. [42], exhibited a sensitivity of 35.2 mV per decade of cholesterol concentration, whereas the CuO nanowires-based sensor developed by Ibupoto et al. [43] demonstrated a sensitivity of 33.88 mV/decade of cholesterol concentration; comparable sensitivities have been reported by similar platforms, such as the electrochemical cholesterol sensor based on cholesterol oxidase andMoS_2_-AuNPs modified glassy carbon electrode (38 mV per decade) [44] or the modified Au nanowires-electrochemical biosensor based on MEMS (microelectromechanical) micro-fluidic platform (42 mV per decade) [45]. The sensitivity improvement observed herein may be largely attributed to the presence of the lipid film. As cholesterol and the lipids of the film share similar chemistry, cholesterol molecules are strongly attracted to the surface of the lipid film resulting in both increased concentration of the analyte at the vicinity of the enzyme and phase alteration of the lipid layer to a more packed structure (i.e., from liquid crystalline to gel) [40,46]. While the former increases sensitivity the latter enhances signal amplification. In effect, the biochemical interaction is optimised, allowing for a comparison between the two systems at the macro-level.

### 3.1. Sensor Construction

Fabrication of electrodes share similar demands on expertise and costs; the latter considered for laboratory procedures and research purposes only. The preparation of graphene nanosheets is a longer process and extra care should be exercised to avoid aggregated graphene that tends to weaken the advantage of the large specific surface area. Still, the material is more easy to handle than nanowalls. The scalability of both processes has yet to be determined.

In general, the graphene-based system performed better than ZnO nanowalls. Although in both systems a successful sensor has been constructed in >98% of the attempts, the electrochemical response of the graphene-bioelectrode was more stable and reproducible. The graphene noise levels were consistently lower by 15% and the variability observed ranged between 2.5% and 3.1% compared to 2.1%–4.8% that the ZnO platform provided (Table 1). The noise level is a critical sensor parameter as the detection limit is determined as the signal with a magnitude three times the noise level (S/N = 3).

Both systems exhibit a high surface to volume ratio. The graphene nanosheets are excellent hosts for the lipid molecules, providing some degree of anchoring [22] that assures reproducibility of construction and low noise. The ZnO nanowalls, on the other hand, possess successive layers of positive and negative ions along its nonpolar plane [40], which enhances further the adsorptivity of the enzyme, thus giving a capability for high sensitivity.

### 3.2. Sensor Characteristics and Performance

**Figure 3:**
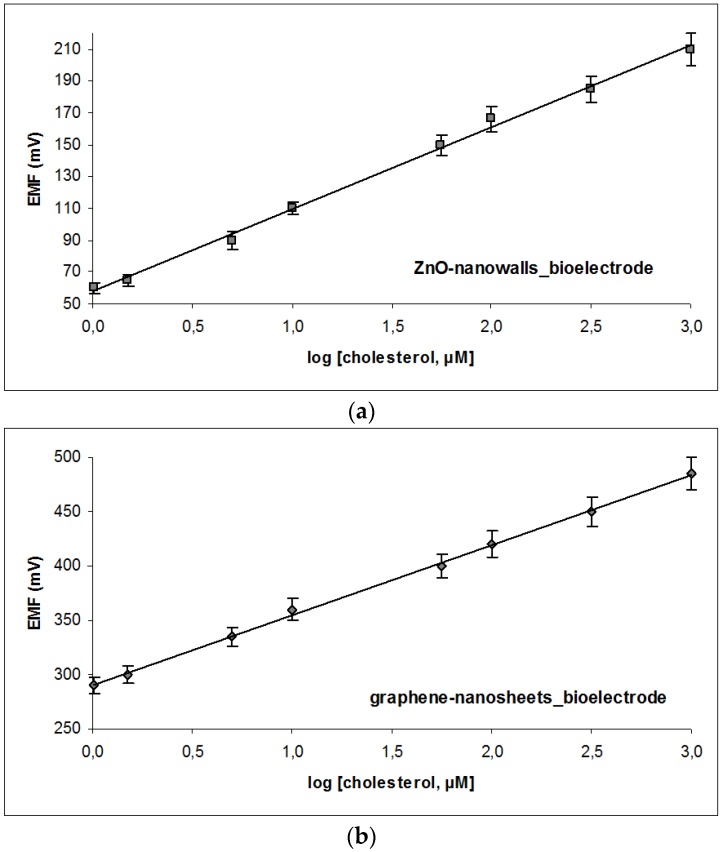
Calibration curve for cholesterol detection using the miniaturized potentiometric sensor: cholesterol oxidase incorporated in polymerized lipid films on ZnO nanowalls (**a**) or graphene nanosheets (**b**). Experimental conditions: pH 7.4 (PBS); temperature: 25 °C; reference electrode: Ag/AgCl. Error bars denote standard deviation (*n* = 38 for the ZnO-based sensor and *n* = 36 for the graphene-based sensor).

A two electrode system (lipid membrane/working bioelectrode and a standard Ag/AgCl reference electrode) has been utilized to measure the potentiometric electrochemical response of the sensors. A quick electrochemical output response, as a function of the time, of the biosensors has been observed over the whole range of the concentrations with ~95% of the steady stable voltage signal at ~5 s. All the above measurements have been performed on the bases of relative potential difference (EMF) among working electrode and the reference electrode [39]. The graphene-based system exhibited a slightly higher sensitivity slope curve (64 mV per decade of cholesterol concentration) as compared to the ZnO platform (57 mV per decade of cholesterol concentration) (Figure 3). Although the working range of both formats is comparable (Table 1), the detectability of the graphene-nanosheets is five times lower than the detection limit of the ZnO nanowalls alternative. As evident, the lower noise levels and the more efficient coupling of the lipid film with the electrode proved more advantageous for the enzyme-based system; it is worthwhile noting that in the case of immunosensors, the benefits might be reversed. Sensor response towards a given analyte concentration is the net result of many physical and chemical contributing factors one of the most critical ones being protein activity. Judging merely from macroscopically (from the signals attained), graphene-systems seems to aid lipid packing transformations; on the other hand, the electroconductivity of ZnO-nanostructures is superior and even more suitable for other biosensor formats.

Enzyme loading could not be increased in any of the two systems. The 15-μL aliquot used corresponded to ca. 7.5 U of enzyme. Further increase of enzyme loading on the lipid film did not result in any considerable decrease of the detection limits or sensitivity improvement, although a 0.5 U increase resulted in 17% higher noise levels in ZnO-based systems.

**Figure 4:**
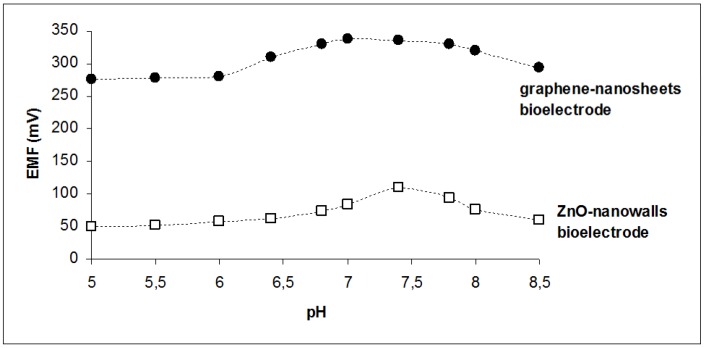
The response of the miniaturized sensors to 5 × 10^−6^ M cholesterol over the pH range 5.5–8.5.

Since the activity of cholesterol oxidase is affected by the pH of the PBS, the performance of the sensors was investigated in the pH range of 5.5–8.5. As shown in Figure 4, ZnO seems to be more sensitive to pH changes, with a clear optimum at pH 7.4. The graphene-based sensor recorded its maximum response at pH 7.0, although the responses obtained between 6.8 and 7.8 pH values exhibited a variation of ±1.8%, which is comparable with the variation observed during calibration. Thus, measurements were performed at pH 7.4 for both sensors.

Reproducibility and stability of both sensors were comparable. Five different electrodes of each type have been tested within five days and different analysts; the maximum variability observed was 5.8% for the graphene-based system and 5.2% for the ZnO-based system, both at the higher cholesterol concentration tested. One sensor from each type has been validated for three days of continuous operation; the biosensors have been carefully soaked into PBS of pH 7.4 before and after each measurement to remove residual biomolecules from its surface. Good stability and linearity has been achieved at this pH value after three days, with a slope drift of 3.5 mV/decade of cholesterol concentration for the graphene-based platform and 2.8 mV/decade of cholesterol concentration for the ZnO-based sensor. If refrigerated when not in use, both sensors hold good chemical activity for a period of 3–4 weeks (with a steady declining trend of ca. 0.3 mV per decade of cholesterol concentration per day).

Regeneration of the sensors was investigated on the use of infinite dilution allowing desorption by mass action to occur. This method is easily implemented by the use of the continuous flow system in which complexes and products are continuously removed. A trade-off has been established for both electrode types involving regeneration time and flow rate; the higher the flow rate, the faster the regeneration of the platform, but the higher the chance of washing the enzyme off. An optimum has been found at 2.5 mL/min for the graphene-based systems and 1.7 mL/min for the ZnO-based systems; the latter exhibited high noise levels with increased flow rates that prohibited further experimentation.

The maximum number of experiments that could be performed, within an acceptable accuracy level, was about 10 for the graphene system, regardless of the cholesterol concentration. Consecutive analyses dropped to six with the ZnO-based systems, especially at high cholesterol levels.

The results from the interference studies were also comparable for both electrode types; the only noticeable interference (ca. 7.5% on signal magnitude) was observed from high albumin levels (5 g/L). The lipidic content of human biofluids may also interfere with the sensor’s lipids. Yet, no significant effect was noticed when serum samples were analyzed; possibly, the presence of the protein sterically hinders lipid–lipid fusion [46].

Serum samples have been analyzed using both systems and the results were compared with those provided from a hospital blood analyzer. The samples were diluted to PBS before measurement. As shown in Table 2, the results from both electrodes were satisfactory and quite close to the analyzer values. Yet, the percent relative error was higher in the case of ZnO-based systems.

## 4. Conclusions

Cholesterol oxidase incorporated in polymerised lipid films have been coupled herein with ZnO nanowalls and graphene nanosheets in a comparative study. The results indicate that the latter seem more suitable for handling catalytic events, slightly better in reliability for real sample measurements and easier to handle for sensor construction. Based on macro-scale data, both materials exhibit comparable sensor longevity and reproducibility while they do not hamper biochemical kinetics. Notwithstanding, more in-depth investigation of the micro-scale parameters (kinetics, local morphology, dielectric properties, etc.) are necessary, before reliable conclusions can be drawn.

The morphology of the lipid membrane mount on the transducer should be characterized by electron microscopy in order to investigate the degree and extent of encapsulation. In perspective, a more detailed investigation of the effect of the graphene or ZnO electrode on the structure of the enzyme-lipid membrane recognition assembly should be performed, using structural measurements (for example, see [47] and references stated therein).

In a broader sense, the ability of fast and simple construction of customisable protein-based biosensing detectors may revolutionise real-time environmental monitoring or personalised self-monitoring. Easily handled, economic, or cost-effective materials with a well-studied and feasible market trajectory may push biosensor science into the market. Graphene technology slightly precedes other nanostructures at technology roadmaps, although more studies are required to evaluate their suitability and applicability.

## Figures and Tables

**Table 1:** Metrological characteristics of graphene-based and ZnO-based cholesterol oxidase biosensors.

	Graphene-Based Sensor	ZnO-Based Sensor
Noise level	29 ± 1.4 mV	34 mV ± 2.3 mV
Equation of calibration	*E_Volts_* = 64.003 × log [*cholesterol*] + 84.861	*E_Volts_* = 54.560 × log [*cholesterol*] + 61.348
	*r*^2^ = 0.9987, [*cholesterol*] in μM	*r*^2^ = 0.9989, [*cholesterol*] in μM
Sensitivity	64 mV/decade of concentration	57 mV/decade of concentration
Variability of response	2.5%–3.1%	2.1%–4.8%
Working range	1 × 10^−6^–1 × 10^−3^ M	1 × 10^−6^–1 × 10^−3^ M
Detection limit	1.08 × 10^−6^ M	5.56 × 10^−6^ M
pH opt	6.8–7.8	7.4

**Table 2:** Comparative performance of graphene-based and ZnO-based cholesterol oxidase biosensors for serum analyses.

Sample Nr.	Hospital Analyser Cholesterol Value	Graphene-Nanosheet Enzyme Sensor		ZnO-Nanowalls Enzyme Sensor	
	mg/dL	mg/dL	% rel. Error	mg/dL	% rel. Error
1	100	98	–2.00%	104	+4.00%
2	116	120	+3.45%	118	+1.72%
3	155	150	–3.23%	153	–1.29%
4	180	185	+2.77%	189	+5.00%
5	201	208	+3.48%	190	–5.47%
6	220	214	–2.72%	230	+4.55%
